# Experimental Study of Direct Laser Deposition of Ti-6Al-4V and Inconel 718 by Using Pulsed Parameters

**DOI:** 10.1155/2014/841549

**Published:** 2014-01-30

**Authors:** Kamran Shah, Izhar Ul Haq, Shaukat Ali Shah, Farid Ullah Khan, Muhammad Tahir Khan, Sikander Khan

**Affiliations:** ^1^Institute of Mechatronics Engineering, University of Engineering & Technology, Peshawar 25100, Pakistan; ^2^Mechanical Engineering Department, University of Engineering & Technology, Peshawar 25000, Pakistan

## Abstract

Laser direct metal deposition (LDMD) has developed from a prototyping to a single metal manufacturing tool. Its potential for creating multimaterial and functionally graded structures is now beginning to be explored. This work is a first part of a study in which a single layer of Inconel 718 is deposited on Ti-6Al-4V substrate. Single layer tracks were built at a range of powder mass flow rates using a coaxial nozzle and 1.5 kW diode laser operating in both continuous and pulsed beam modes. This part of the study focused on the experimental findings during the deposition of Inconel 718 powder on Ti-6Al-4V substrate. Scanning electron microscopy (SEM) and X-ray diffraction analysis were performed for characterization and phase identification. Residual stress measurement had been carried out to ascertain the effects of laser pulse parameters on the crack development during the deposition process.

## 1. Introduction

Laser direct metal deposition (LDMD) has traditionally been used to build small prototypes and in various applications like component repair [1] and short-run component fabrication [2]. The use of LDMD in the aerospace industry is ever increasing; LDMD of titanium and nickel based alloys is extensively applied to the manufacture of small components and to adding details to large parts [3].

Use of multifunctional materials with significant physical properties difference has seen a growing interest in the past few decades. The functionally graded materials (FGMs) concept has been extended to a variety of materials in various applications [4]. In FGM, composition and properties are customized to suit specific engineering applications [5]. The development of FGMs by laser direct metal deposition has received much attention [6, 7]. The freedom to selectively deposit different elemental powders at discrete locations using multiple powder feeders makes LDMD well suited to the fabrication of FGMs.

Deposition of dissimilar materials is qualitatively different from similar material deposition for the following reasons: (1) thermophysical properties of the substrate and clad materials are, in general, different, and this difference can influence the heat transfer during deposition; (2) magnitude of stress differs due to the difference in thermal expansion/contraction properties of the two or more materials; (3) composition becomes a parameter that can vary over a wide range across the melt pool and, consequently, there does not exist a single liquidus isotherm that defines the solid-liquid interface. All layered fabrication processes based on melt deposition, such as laser deposition process, suffer from a residual stress in the final part due to the large temperature gradients between the hot melt zone and the cold substrate [8]. For this reason, there has been very limited work on the deposition of different materials such as nickel and titanium. To avoid sudden change in the composition, a graded structure of Ti-6Al-4V and Inconel 718 was carried out by Domack and Baughman [9] using a laser engineered nest shape (LENS) system. The laser used was in a continuous wave mode and powder was fed as premixed powder blends, with the composition adjusted in steps of 10 percent by volume from 100 percent Ti-6Al-4V to 100 percent Inconel 718. Macroscopic cracks formed before the full transition from Ti-6Al-4V to Inconel 718 were achieved but the cause of these was not investigated in any detail in the reported work. In another detailed study of a titanium and nickel graded structure by laser deposition, Lin et al. [10] investigated the solidification behaviour and phase evolution of Ti-6Al-4V and Rene 88 DT. All proportions of the constituent alloys were not investigated; the compositional gradient tested ranged from 100 percent Ti-6Al-4V to Ti-6Al-4V with 38 percent Rene 88 DT. In all of the previous studies on nickel and titanium, the effects of generation of transient/residual stresses have largely been ignored.

To date, the generation of residual stresses has received relatively little attention. Few authors [11–14] have used neutron diffraction techniques in stainless steel and Inconel 718 laser deposited thin walls and thick pillars. X-ray diffraction technique [15, 16] can also be used to measure residual stresses. When using conventional X-ray diffraction techniques, depth of penetration of the X-ray beam, based on the use of standard copper, iron, chromium, and cobalt anodes, is limited to (3 ± 10 mm) and hence only residual stresses along the surface region can be determined accurately. To obtain stresses at greater depths, removal of material by chemical etching or electric discharge machine (EDM) is necessary.

Previous studies show that functional grading of titanium and nickel alloys cannot provide a complete solution to the problem of stress cracking and hence, in this work, a different approach has been investigated. Here, instead of gradual change in the composition of the Ti-6Al-4V and Inconel 718, deposition of pure Inconel 718 on top of Ti-6Al-4V substrate has been carried out by employing two different modes of laser beam, continuous and pulsed, and different pulse parameters. These two modes produce different thermal gradients which could reduce the transient and hence the residual stresses in the final part. In addition, various powder mass flow rates have been tested to investigate the effect of mass addition on the susceptibility to stress cracking.

## 2. Experimental Procedure

**Figure 1:**
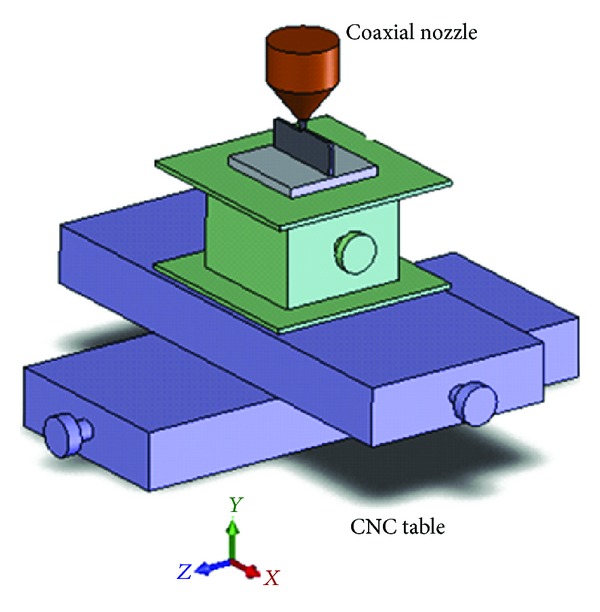
Schematic of the LDMD process.

A series of experiments were performed to investigate single layer laser deposition of Inconel 718 on Ti-6Al-4V substrate. Schematic of the process is shown in Figure 1. Two sets of samples for each parameters combination were used in order to analyze the effect of diode laser pulse parameters and powder flow rate, with varying pulse length and period. Inconel 718 powder was deposited on a Ti-6Al-4V substrate. The substrates were machined into 48 × 50 × 11 mm rectangular coupons. These substrates were first sand blasted in a Guyson sand blaster and then degreased using ethanol to aid deposit adhesion. In this study, 9 samples were produced. A 1.5 kW Laserline LDL 160–1500 diode laser, producing approximately equal amounts of 808 and 940 nm laser radiation operated in either continuous or pulsed mode, was used to carry out the experiments. The beam size was experimentally measured by exposing an infrared detector card with a sensitivity of 1.75 × 10^−9^ W/mm^2^ to the pilot beam (20 mW) of the diode laser and was found to be 1.67 mm with top hat (approximately uniform) power distribution.

The absorptivity of diode laser wavelengths (808 and 940 nm) was measured using a SD2000 fibre optic spectrometer which was 45~51%. The substrate was tightly secured on a clamp and then mounted onto a 3-axis position and motion control system. Inconel 718 powder was gas atomised, giving particles with approximately spherical morphology, and had a powder size distribution of 53–150 *μ*m. A disc-type powder feeder was used to deliver them to the nozzle. The deposition area was also shrouded by an Argon gas flow from a coaxial nozzle directed at the melt pool. The gas pressure for all the experiments was set at 5 bars. A velocity of 4 mm/s was maintained during the deposition of all the samples.

Parameters were varied specifically to enable the study of the effect of powder flow rate and the duty cycle. A mean power of 600 W was used as established from previous experiments. This provided a specific energy appropriate for the deposition of both Inconel 718 and Ti-6Al-4V at the chosen traverse speed and beam area and was kept constant throughout. The experimental parameters used are shown in Table 1.

After the experiments, uncracked samples were held in a fixture on HURCO 425 Mark 2 sink type spark erosion electric discharge machine (EDM). Samples were longitudinally machined till they reached the middle of the clad by using copper as a tool electrode.

After machining the samples, residual stresses on one set of uncracked deposited tracks (S1–S3) were measured using X-ray diffraction technique, based on sin 2*ψ* method [17] which uses the planes of crystal lattice as an atomic scale “strain gauge.” X-ray residual stress analysis is based on the angular shift of diffraction lines due to stresses [18].

A Bruker AXS D8 Discover laboratory X-ray diffractometer was used. This equipment has a polycapillary optics X-ray beam and general area detector diffraction system (GADDS) detector collecting backscattered X-ray Debye-Scherrer cones. An iron anode X-ray tube (*λ* = 1.9360 Å) at 30 kV and 20 mA was used and the 311 diffraction peak was recorded at a “2*θ*” angle of about 128°. 10 *ψ* values, from 0 to 45 degrees, were used for each point, giving a data collection time of 200 seconds for each *θ* value. Young's modulus value of 200 GPa and Poisson's ratio value of 0.30 [19] were used to calculate residual stresses.

X-ray diffraction phase analyses were carried out using a Philips X'Pert MPD X-ray diffractometer with a Cu anode to provide radiation of 1.5405 Å wavelength. For each sample, the deposited tracks were separated from the substrate and the interface was scanned between 2*θ* angles of 0 and 85 degrees with a 30-second scan step time. To perform the X-ray diffraction phase analysis, samples 1, 2, and 3 were broken from the substrates, forcefully if necessary. X-ray diffraction phase analyses were then carried out on the bottom side of clad and on the top of the substrate where individual tracks were deposited. After stress analysis, the other set of uncracked samples were transversely sectioned and mounted in Struers epoxy resin. All samples were polished to Ra = 1 *μ*m using standard metallographic techniques. The samples were electrolytically etched in 10% oxalic acid to reveal the microstructure. These microstructures were then evaluated using a Hitachi High Technologies, S-3400N Type I, 0–30 kV scanning electron microscope.

## 3. Results

### 3.1. Macrostructure and Visible Cracking

**Figure 2:**
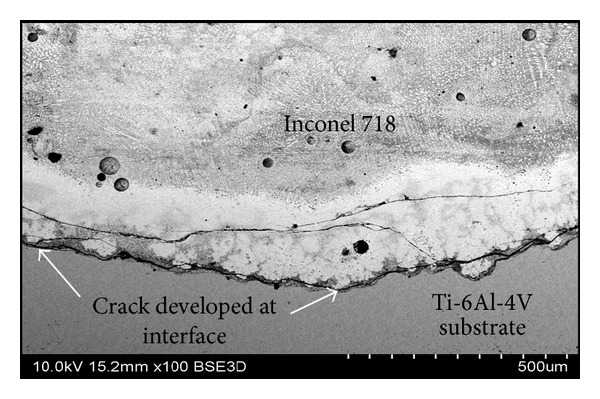
Cracked interface between Ti-6Al-4V and Inconel 718 in sample 4.

Examination of the final Ti-6Al-4V and Inconel 718 structures showed that only three out of the nine samples were crack-free. Sample 1 (0.4 duty cycle, low powder mass flow rate), sample 2 (0.7 duty cycle, low powder mass flow rate), and sample 3 (continuous wave, low powder mass flow rate) showed no signs of cracking. All other samples developed severe cracks initiated at the interface of the clad and the substrate. Cross section of cracked interface of sample 4 is shown in Figure 2.

### 3.2. Phase Analysis Using X-Ray Diffraction

**Figure 3:**
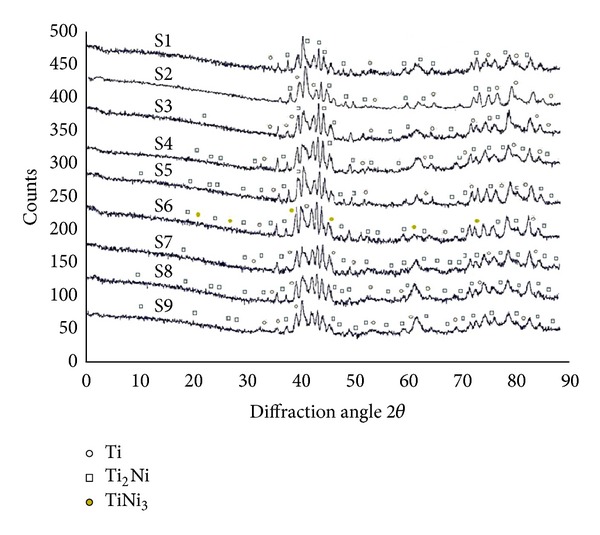
XRD diffraction patterns showing the presence of various phases.

Different phases constitute the microstructure of the melt pool and these are identified from the composition analyses using X-ray diffraction. In this work, despite the small size of the samples, clear spectra with well-defined diffraction peaks were obtained.  Figure 3 shows X-ray diffraction spectra for all the samples. The XRD patterns for all the samples look very similar except in sample 6 which reveals the presence of TiNi_3_. All these results indicate the presence of Ti_2_Ni and Ti in all of the samples. Quantification of the phases present was not possible.

### 3.3. Residual Stress Results

**Figure 4:**
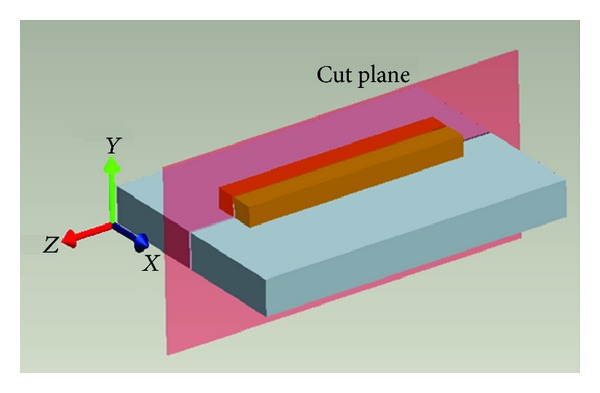
Substrate and clad geometry.

**Figure 5:**
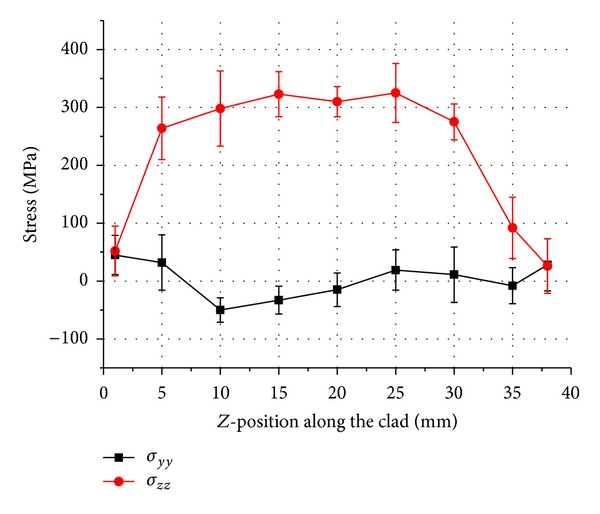
Stress result in sample 1, 0.4 DC pulsed laser deposition.

**Figure 6:**
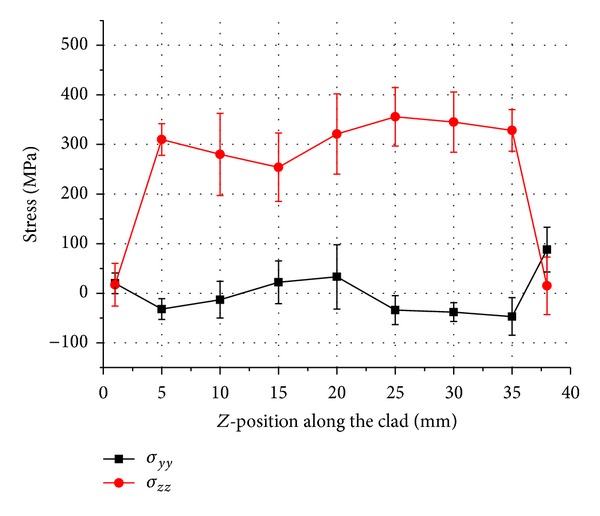
Stress result in sample 2, 0.7 DC pulsed laser deposition.

**Figure 7:**
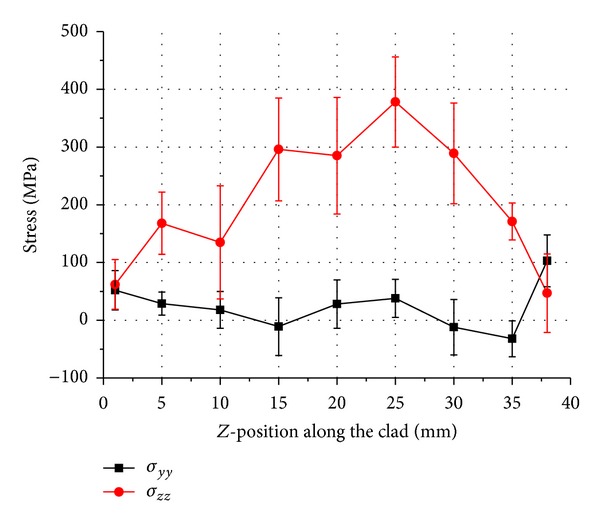
Stress result in sample 3, continuous wave laser deposition.

Uncracked samples were machined in a *y*-*z* plane as shown in Figure 4. Line scans were carried out to measure vertical “*σ*_*yy*_” and longitudinal “*σ*_*zz*_” residual stresses in the middle of the clad and along the track length of uncracked samples 1–3. The measured distributions are plotted for three different duty cycle depositions in Figures 5, 6, and 7, respectively.

Figures 5–7 show an approximately parabolic distribution of residual stresses in the *z*-direction with a maximum calculated stress close to the centre position. There is more variation in stress values in case of pulsed deposition as compared to the continuous wave deposition. Increase in tensile stresses in longitudinal direction with the increase in duty cycle is also noted. A maximum tensile stress “*σ*_*zz*_” value of 583 MPa is observed in the continuous wave deposition midway along the clad and residual stress of approximately 0 MPa at the ends of the tracks. Stress “*σ*_*yy*_” is seen to be compressive along the clad length in all three samples, with the exception of ends of the deposited clad where low levels of tensile residual stresses are obtained.

## 4. Discussion

Factors which can cause deposited clads to crack can be divided into metallurgical and mechanical factors. Metallurgical factors mainly concern phase relationships, while mechanical factors are related to stress behaviour.

Considering metallurgical factors, XRD results show the presence of Ti_2_Ni and TiNi_3_ in all of samples whether cracked or uncracked. Ti_2_Ni and TiNi_3_ are recognized as one of the brittle phases responsible for cracking in welding and their presence in all samples suggests that an appropriate selection of laser parameters may not be sufficient to avoid the production of such intermetallics.

Taking into account the mechanical factors, stresses can be generated by constrained elastic expansion or contraction due to transient temperature gradients, and thermal expansion coefficient mismatch, and changes in specific density due to solid phase transformations. The amount of heat input determines the cooling rate, which is inversely proportional to the square of the melt pool length [20]. Thermal strains caused by high cooling rates can increase the crack initiation rate, but higher thermal gradients result in a rapid cooling rate and as, has been shown previously in case of a weld, can also reduce the grain size to increase solidification and crack resistance [21].

Results presented here suggest that the trend in residual stresses generated during the deposition of Inconel 718 by either pulsed or continuous beam does not vary significantly apart from the continuous wave deposition which exhibits a comparatively higher residual stress in the longitudinal direction. This can be explained by the fact that the cooling rate for the continuous wave deposition samples is higher as shown by the clad width. Consequently, a larger proportion of residual stress in longitudinal direction is observed as compared to the shorter pulse length or low duty cycle samples. This kind of conclusion is also reported by Moat et al. [14] who found large tensile longitudinal stresses at the mid position of the wall. The vertical stresses “*σ*_*yy*_” are tensile near the vertical free surfaces which are balanced by the compressive stresses in the interior of the track.

It is observed that the stresses developed during low powder feed rate deposition (continuous and pulsed) are less than the yield strength of either of the two materials (Inconel 718 and Ti-6Al-4V) and the phases which are present in the samples are also similar. The measurement of residual stresses in this work is limited only to low powder deposition. It is necessary, however, to establish the transient behaviour of stresses generated during the medium and high powder mass flow rate deposition. Numerical modelling of the deposition process may provide the best means of estimating these stresses.

## 5. Conclusions

Various pulse parameters for a diode laser have been used to exploit the effects of direct laser metal deposition of Inconel 718 on Ti-6Al-4V substrate. The driver for this study is to understand and analyse the deposition of Inconel 718 and Ti-6Al-4V by examining the stress fields and phases evolved during LDMD. XRD techniques have been utilized for the residual stresses and phases generated during the deposition process. It has been found that two brittle phases, that is, Ti_2_Ni and TiNi_3_, are produced during the deposition of all of the samples. It has also been observed that high longitudinal tensile stresses are developed along the tracks during the deposition process and increase with the duty cycle. The effect of duty cycle on residual stresses in low powder deposition process is, nevertheless, inconclusive and makes it difficult to explain the crack initiation.

## Figures and Tables

**Table 1:** Experimental parameters used in the experiment.

Sample no.	Peak power (W)^A^	Pulse length (ms)	Period (ms)	Duty cycle^B^	Powder mass flow rate (g/s)
1	1500	20	50	0.4	0.358
2	857	35	50	0.7	0.358
3	600	—	—	1	0.358
4	1500	20	50	0.4	0.586
5	857	35	50	0.7	0.586
6	600	—	—	0.1	0.586
7	1500	20	50	0.4	0.674
8	857	35	50	0.7	0.674
9	600	—	—	1	0.674

^A^Mean power = 600 W in all cases; ^B^duty cycle = pulse length/period.
